# The look ahead trace back optimizer for genomic selection under transparent and opaque simulators

**DOI:** 10.1038/s41598-021-83567-5

**Published:** 2021-02-18

**Authors:** Fatemeh Amini, Felipe Restrepo Franco, Guiping Hu, Lizhi Wang

**Affiliations:** Department of Industrial and Manufacturing Systems Engineering, Iowa State University, Ames, IA 50011 USA

**Keywords:** Biotechnology, Computational biology and bioinformatics, Mathematics and computing

## Abstract

Recent advances in genomic selection (GS) have demonstrated the importance of not only the accuracy of genomic prediction but also the intelligence of selection strategies. The look ahead selection algorithm, for example, has been found to significantly outperform the widely used truncation selection approach in terms of genetic gain, thanks to its strategy of selecting breeding parents that may not necessarily be elite themselves but have the best chance of producing elite progeny in the future. This paper presents the look ahead trace back algorithm as a new variant of the look ahead approach, which introduces several improvements to further accelerate genetic gain especially under imperfect genomic prediction. Perhaps an even more significant contribution of this paper is the design of opaque simulators for evaluating the performance of GS algorithms. These simulators are partially observable, explicitly capture both additive and non-additive genetic effects, and simulate uncertain recombination events more realistically. In contrast, most existing GS simulation settings are transparent, either explicitly or implicitly allowing the GS algorithm to exploit certain critical information that may not be possible in actual breeding programs. Comprehensive computational experiments were carried out using a maize data set to compare a variety of GS algorithms under four simulators with different levels of opacity. These results reveal how differently a same GS algorithm would interact with different simulators, suggesting the need for continued research in the design of more realistic simulators. As long as GS algorithms continue to be trained in silico rather than in planta, the best way to avoid disappointing discrepancy between their simulated and actual performances may be to make the simulator as akin to the complex and opaque nature as possible.

## Introduction

Plant breeders have been relying primarily on the phenotypic selection (PS) to select breeding parents that maximize the genetic gain and increase grain yield^1^. However, multiple studies have demonstrated that the current annual global crop yield growth rates are below the 2.4% growth rate required to meet projected crop demand in 2050^2,3^. Genomic Selection (GS), pioneered by Meuwissen et al.^4^, has been widely accepted as a game changer in animal and plant breeding^5^. Contrary to PS, GS allows breeders to identify superior individuals in the breeding population using genotypic in addition to phenotypic data.

Rapid development of genotyping and phenotyping technologies alongside deployment of modern computational capabilities has led to increasingly comprehensive databases and intelligent algorithms, further enabling the application of GS. Next-generation sequencing has enabled fast genome-wide marker mapping at low costs, increasing the availability of high-density marker information that improves model accuracy^1,6^. Furthermore, high throughput phenotyping has allowed for rapid and accurate collection of phenotypical data via non-invasive imaging^7^. The use of these novel phenotyping and genotyping methods has increased the availability of high-quality datasets required to create accurate GS models^8^. Lorenzana and Bernardo^9^ demonstrated that cumulative response from three cycles of genome wide biparental GS via best linear unbiased prediction in maize would yield 1.5 times more genetic gain than that of a PS cycle. Heffner et al.^10^ found that the overall GS prediction accuracy for thirteen agronomic traits was 14% higher than that of PS.

The effectiveness of GS has been found to rely on the accuracy of genomic prediction^11^, especially in complex traits due to the predominance of epistatic effects^6^. González-Camacho et al.^12^ found ridge regression and Bayesian models to perform exceptionally well when additive traits are modeled. A study conducted by Crossa et al.^6^ further corroborated these findings by comparing various linear and nonlinear models across multiple traits and environmental conditions using maize and wheat datasets. Under low-density marker conditions, Bayesian Lasso yielded the highest prediction accuracy for additive traits male and female flowering. However, when high-density markers were used, reproducing kernel Hilbert space (RKHS) slightly outperformed Bayesian Lasso. This could be attributed to RKHS’s ability to better capture epistatic interactions under high-density marker conditions^6^. More recently, Shikha et al.^13^ conducted a similar study, where the prediction accuracy of seven different prediction models was evaluated for multiple traits in different environments. Their study found that Bayes B, a linear approach, yielded the best overall prediction accuracy, closely followed by RKHS. These results suggest that accuracy of genomic prediction models may be sensitive to trait type (additive or complex), environmental effects, and marker density. Although advanced technologies have facilitated the data acquisition in GS, designing an appropriate prediction model to handle big data remains challenging. Recently, Amini and Hu^14^ designed a two-layer feature selection algorithm that can reduce the data dimension while maintaining the prediction accuracy.

Although genomic prediction accuracy plays an essential role in achieving genetic gain, few studies have addressed how improved selection and mating strategies can provide room for higher and faster genetic gain. Prior to Goddard^15^, truncation selection was used as the default strategy for selecting breeding parents, as genetic gain was treated as a consequence of implementing genomic prediction, whose accuracy was found to be positively correlated with genetic gain^16^. Goddard^15^ used the weighted genomic estimated breeding values (WGEBV) as a variation of the conventional genomic selection (CGS) approach by Meuwissen et al.^4^, where rarer alleles were given higher weights to increase their frequency and the long term response. Daetwyler et al.^17^ proposed the optimal haploid value (OHV) for selecting breeding parents, focusing on haploid selection to generate an elite fixed line. Goiffon et al.^18^ presented optimal population value (OPV), a population-based selection strategy, where the merit of a breeding population is evaluated based on the complementarity of the group rather than the summation of individual parent’s contributions. More recently, Moeinizade et al.^19^ proposed the look ahead selection (LAS) approach, which attempts to improve genetic gain by maximizing the probability of producing elite progeny by a target deadline. LAS has been shown to outperform previous selection and mating strategies due to its unique capability to anticipate, or look ahead, how decisions made in the current generation would affect the progeny in the target generation. In “The LATB optimizer”, we propose a new approach, the look ahead trace back (LATB) algorithm, to further improve the performance of LAS in terms of genetic gain, especially with imperfect prediction of allele effects.

Besides selection and mating strategies, another important component that has not received enough attention in the GS literature is the simulator that we use to evaluate the performance of selection algorithms. Previously, GS approaches have been tested in transparent simulation settings, in which full genotype data and additive allele effects are assumed to be known, and no dominance effect, epistases, or genotype by environmental interactions are explicitly captured. However, such transparent simulators may not realistically reflect the opaque and complex nature that we live in, where selection and mating decisions are made based on partially observable information under uncertainty. To alleviate the discrepancy between simulation and nature, we propose our design of an opaque simulator in “Opaque simulator”, which simulates nature with high dimensional data of assumed ground truth with multiple sources of uncertainty that are genetically meaningful, and only a subset of which is observable by the GS algorithms. We conducted a comprehensive computational experiment in “Computational experiments” to test the performances of four GS algorithms under four different simulators, which produced insightful results.

## Materials and methods

**Figure 1:**
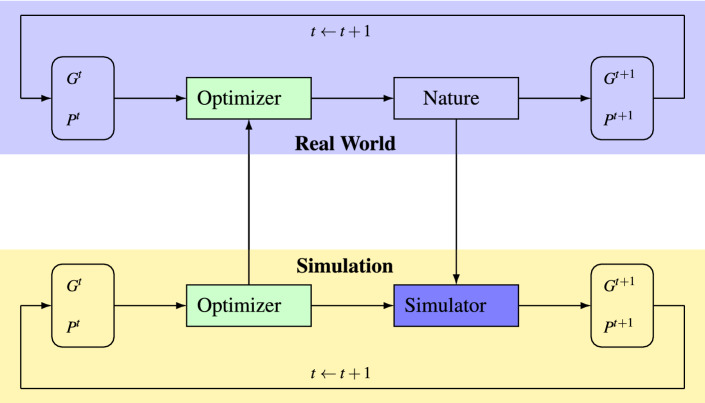
Roles of simulator and optimizer in genomic selection.

Numerous decisions must be made, by either experienced breeders or automated tools, in multiple stages of a breeding program under a great deal of uncertainty. The effectiveness of these decisions has overarching and long-lasting implications to the success of the breeding program. Historically, breeders used methods such as phenotypic selection, progeny testing and BLUP for decades^20–22^. In the era of data-driven molecular breeding, more emerging tools such as high performance computing resources and sophisticated algorithms are becoming accessible to help breeders accelerate genetic gains. However, the time-consuming, resource-intensive, and high-risk nature of the breeding process, especially considering multi-traits breeding, makes it prohibitive to design, validate, and train the algorithms directly during the actual breeding process^23,24^. Therefore, an in silico “simulator” that mimics nature reasonably well becomes critical for training and evaluating the “optimizer” in GS research, as illustrated in Fig. 1. The optimizer determines the crosses to make based on historical genotype and phenotype data, nature determines the next generation genotype as a result of the crosses and produces the next generation phenotype as a result of the genotype and environment interactions, and the simulator attempts to mimic how nature works. In the following subsections, we propose a new design of simulator and a new algorithm as the optimizer.

### Transparent simulator

In conventionally used simulators^17–19^, almost all information is known to the optimizer, so we refer to these as transparent simulators. To make such a simulator, historical genotype ($G^0 \in \mathbb {B}^{n^0, p, 2}$) and phenotype ($P^0 \in \mathbb {Q}^{n^0}$) data are used to estimate the allele effect vector $\beta \in \mathbb {Q}^P$ for a linear model $P^0 = G^0 \beta$^6,12,25,26^, and the trained parameter $\beta$ is then used in the simulator. Here, $n^0$ is the number of individuals in the initial population, *p* is the number of markers, and the third dimension in $G^0$ represents the two chromosomes in a diploid species. Oftentimes parameter $\beta$ is also passed along to the optimizer as known information^17–19^. Function $h(G^t|r, S)$ simulates the creation of the $(t+1)$st generation genotype from the *t*th generation according to the Reproduce function used by Han et al.^27^, with $r \in [0, 0.5]^{p-1}$ being the recombination frequencies vector and *S* denoting the selection decision from an optimizer, which specifies the breeding parents selected from $G^t$.

### Opaque simulator

**Figure 2:**
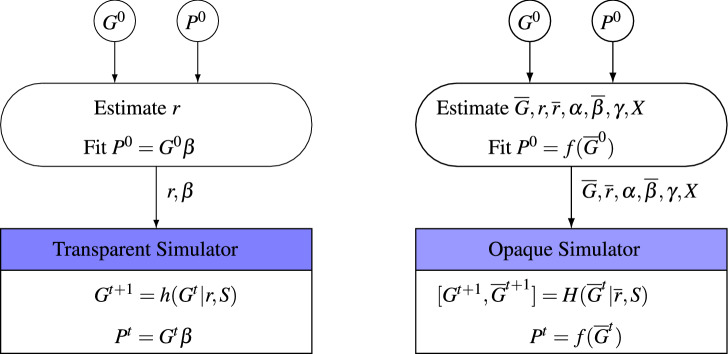
Designs of transparent (left) and opaque (right) simulators.

The proposed simulator attempts to serves as a more realistic representation of nature. We call it an opaque simulator because only partial information is observable to the optimizer. Figure 2 illustrates the differences between a transparent and an opaque simulator. Details of an opaque simulator are described as follows.

**Figure 3:**
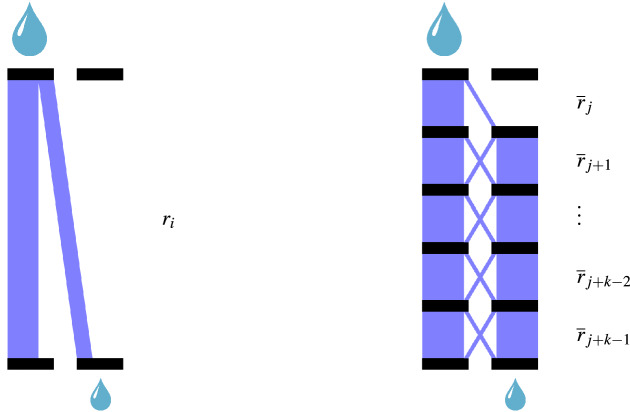
Illustration of relationship between recombination frequencies $r_i$ and $\overline{r}_j, ..., \overline{r}_{j+k-1}$ using a water pipe model^27^.

- The opaque simulator treats the observed genotype data as samples at a subset of marker loci, denoted as $\mathcal {P}$, of an assumed whole genome loci, denoted as $\mathcal {\overline{P}}$. The assumed whole genome is constructed by augmenting the observed genotype data of the initial generation, $G^0 \in \mathbb {B}^{n^0, p, 2}$, to a much higher-dimensional space. The resulting genotype, $\overline{G}^0 \in \mathbb {B}^{n^0, \overline{p}, 2}$, contains $G^0$ at the marker loci, i.e., $\overline{G}^0_{:, \mathcal {P}, :} = G^0$. The whole genome will be used throughout the breeding process inside the opaque simulator, which will evolve over time as a result of recombination, whereas only the genotype at the marker loci $\mathcal {P}$ is observable to the optimizer in each generation.
- We construct the recombination frequencies vector $\overline{r} \in [0, 0.5]^{\overline{p}-1}$ for the assumed whole genome based on the estimated recombination frequencies vector $r \in [0, 0.5]^{p-1}$ at the marker loci $\mathcal {P}$. Suppose two adjacent loci $i, i+1 \in \mathcal {P}$ correspond to two non-adjacent loci $j, j+k \in \mathcal {\overline{P}}$ separated by $k-1$ other loci in between. Given $r_i$, the recombination frequency between loci *i* and $i+1$, the recombination frequencies $\overline{r}_j, \overline{r}_{j+1}, \ldots , \overline{r}_{j+k-1}$ must satisfy the following equations:
  1 $$\begin{aligned} w_{j,1}= & {} 1 \end{aligned}$$
  2 $$\begin{aligned} w_{j,2}= & {} 0 \end{aligned}$$
  3 $$\begin{aligned} w_{l,2}= & {} w_{l-1,1} (1-\overline{r}_{l-1}) + w_{l-1,2} \overline{r}_{l-1}, \forall l \in \{j+1, \ldots , k\} \end{aligned}$$
  4 $$\begin{aligned} r_i= & {} w_{k,2}. \end{aligned}$$
  Mathematically, these equations ensure that the probability of a recombination between two adjacent marker loci *i* and $i+1$ is the same as the probability of a recombination between two non-adjacent marker loci *j* and $j+k$. Intuitively, using the same water pipe model proposed by Han et al.^27^, the two aforementioned probabilities are analogous to the amounts of water coming out of the left and right plumbing systems in Fig. 3 when a unit amount of water is poured into the valve $w_{j,1}$. In Eq. (4), $w_{j,c}$ is the amount of water that comes out of valve *c* of level *j*. The recombination frequency $r_i$ is equal to $w_{k,2}$ because it is the amount of water that comes out from the last layer of valve at column 2 when one unit of water was poured into the first layer of valve at column 1 after *k* layers of redistribution (recombination).
- Similar with function $h(G^t|r, S)$, function $H(\overline{G}^t|\overline{r}, S)$ simulates the creation of the $(t+1)$st generation genotype $\overline{G}^{t+1}$ from the *t*th generation according to the Reproduce function used by Han et al.^27^, with $\overline{r}$ being the recombination frequencies vector and *S* denoting the selection decision from an optimizer, which defines the breeding parents selected from $G^t$.
- Phenotype $P^t$ that corresponds to genotype $G^t$ is determined as follows:
  5 $$\begin{aligned} P^t_i= & {} f(\overline{G}^t_i) = \sum \limits _j \overline{\beta }_j (\overline{G}^t_{i,1,j} + \overline{G}^t_{i,2,j}) \end{aligned}$$
  6 $$\begin{aligned}&+ \sum \limits _j \alpha _j I(\overline{G}^t_{i,1,j} \ne \overline{G}^t_{i,2,j}) \end{aligned}$$
  7 $$\begin{aligned}&+ \sum \limits _k \gamma _k \prod \limits _{j,m} I(\overline{G}^t_{i,m,j} + X_{m,j,k}\ne 1) \end{aligned}$$
  8 $$\begin{aligned}&+ \epsilon _i, \quad \qquad \qquad \forall i. \end{aligned}$$
  Here, $\overline{\beta }_j$ in Eq. (5) is the additive effect of gene *j* in the assumed whole genome; $\alpha _j$ in Eq. (6) is the dominance effect at locus *j*; $\gamma _k$ in Eq. (7) is the epistatic effect of interaction *k*; matrix $X \in \{0, 0.5, 1\}^{\overline{p} \times 2 \times K}$ defines the membership of genes that are involved in the interactions, with $X_{m,j,k}=1$ indicating that gene $(m,j) = 1$ is necessary to trigger interaction *k*, $X_{m,j,k}=0$ indicating that gene $(m,j) = 0$ is necessary to trigger interaction *k*, and $X_{m,j,k}=0.5$ indicating that gene (*m*, *j*) is not involved in the interaction *k*; and $\epsilon _i$ is a random noise, representing environmental effects and other effects not accounted for in the model. Equation (6) means that that dominance effect at locus *j* is triggered if and only if $\alpha _j$ is non-zero and the two alleles are heterozygous. The indicator function $I(\overline{G}^t_{i,m,j} + X_{m,j,k}\ne 1)$ in Eq. (7) means that epistatic effect *k* is triggered if and only if the genotype $G_{i,m,j} = X_{m,j,k}$ for all genes *i* that are involved in the effect *k*. Equations (5)–(8) are essentially overfitting the observed relationship between genotype $G^0$ and phenotype $P^0$ to integrate the dominance and epistatic effects. As a result, there may exist infinitely many solutions to satisfy Eqs. (5)–(8) with $P^0 = f(\overline{G}^0)$, and any one could be used as an opaque simulator as long as the parameters are within a reasonable range. This is because the purpose of an opaque simulator is to reveal how an optimizer might interact with an opaque nature rather than predict how nature will act.

### The LATB optimizer

The “optimizer” in Fig. 1 has two main tasks: prediction and selection. Previous research effort in GS has disproportionately focused on genomic prediction with truncation selection being the default selection strategy.

In recent years, a series of algorithms have been proposed for making more strategic selection decisions. These previous algorithms such as CGS, WGEBV, OHV, OPV, and LAS were designed and tested in transparent simulators, hence their performance under opaque simulators has not yet been tested. A major challenge is the fact that the estimated additive allele effects may no longer be consistent with the true relationship between genotype and phenotype, which is assumed to be non-additive, unknown, partially observable, and noisy under an opaque simulator. These recent algorithms achieved improved genetic gains by strategically combining favorable alleles and removing unfavorable ones; when the accuracy of the estimated allele effects becomes questionable, so does the superiority of these algorithms.

**Figure 4:**
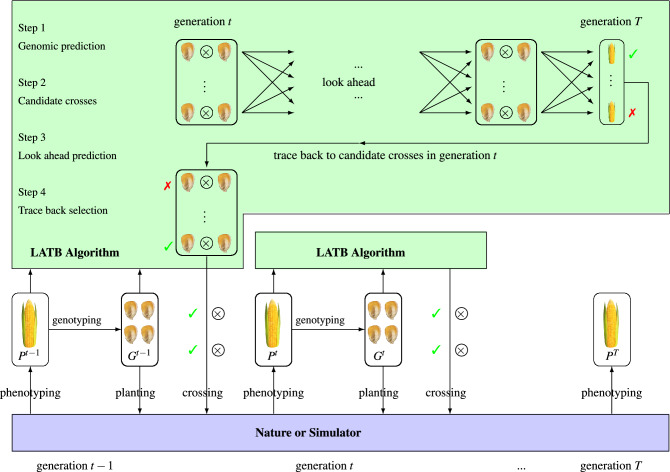
Illustration of how the LATB optimizer interacts with nature or a simulator.

In this section, we present the LATB algorithm as a new optimizer for GS under opaque simulators. This algorithm consists of four major steps, which are illustrated in Fig. 4 and described as follows.

**Step 1: Genomic prediction**

Conventionally, genomic prediction is used to estimate an allele effect vector $\beta$ based on genotype data *G* and phenotype data *P* by fitting a linear relationship $P = G \beta$. In the LATB algorithm, instead of estimating only one $\beta$, we use different algorithms or different hyperparameters of a same algorithm to produce a number of allele effect vectors $\beta ^s, \forall s \in \mathcal {S}$. The purpose is to reduce the chance for the selection to be biased by arbitrary choices in genomic prediction methods rather than statistically significant allele effects. We refer to these vectors as different scenarios. Intuitively, a larger number of scenarios is more likely to enclose the truth.

**Step 2: Candidate crosses**

A large pool of candidate crosses is created, comprised of random crosses between high potential individuals (based on phenotype, genotype, or pedigree). To ensure the quality of the selection, the candidate pool should be large enough to include a variety of crosses subject to computational constraints on time and storage capacity.

**Step 3: Look ahead prediction**

Progeny from the candidate crosses are simulated and estimated using all scenarios of the allele effect vectors, and then the top performers are randomly mated with each other to produce the next generation; this process iterates until the final generation *T*. The purpose of this step is to anticipate the consequences of the candidate crosses, and the multiple allele effect vectors are used to provide a more robust performance assessment, i.e., an individual whose performance is sensitive to $\beta$ is less robust and may be less preferable than another that performs reasonably well under all scenarios.

In essence, this step uses observed genotype *G* and estimated linear function $P = G \hat{\beta }$ (as opposed to the assumed true genotype $\overline{G}$ and phenotype function $P = f(\overline{G})$, which is unbeknownst to the optimizer) to look ahead, or anticipate, the consequences of the candidate crosses in order to identify the optimal set of crosses.

**Step 4: Trace back selection**

We trace all individuals in the final generation of step 3 back to their ancestors in the candidate pool. Let *M* denote the binary relationship matrix between candidate crosses and these individuals, with $M_{c,i}=1$ indicating that individual *i* is an offspring of cross *c* and $M_{c,i}=0$ otherwise. Let $\hat{P}^T_i$ denote the estimated phenotype of individual *i* in the final generation *T* of step 3. Then the selection problem can be formulated as the following integer linear program.

9 $$\begin{aligned} \max _{x,y}&\sum \limits _i \hat{P}^T_i y_i&\end{aligned}$$

10 $$\begin{aligned} {{\,\mathrm{s.t.}\,}}&\sum \limits _c x_c = s&\end{aligned}$$

11 $$\begin{aligned}&y_i - x_c + M_{c,i} \le 1&\forall i, c \end{aligned}$$

12 $$\begin{aligned}&x_c, y_i \text { binary}&\forall i, c. \end{aligned}$$

The objective function (9) is to maximize the phenotypic performance of the individuals that could be produced. Decision variable $y_i = 1$ indicates that individual *i* can be produced (because all its ancestors in the candidate pool have been selected) and $y_i = 0$ otherwise. Constraint (10) means that no more than *s* crosses can be made. Decision variable $x_c = 1$ indicates that cross *c* is made and $x_c = 0$ otherwise. Since only a subset of the candidate crosses will be made, not all individuals from step 3 can be produced. Constraint (11) specifies the relationship among $x_c$, $y_i$, and $M_{c,i}$: individual *i* could not be produced unless all of its founding crosses were made.

## Computational experiments

### Simulator settings

We used a dataset^28^ that consists of around 1.4 million SNPs (single nucleotide polymorphism) from the 369 maize inbred lines of shoot apical meristem (SAM) population distributed across the 10 maize chromosomes. They have been collected using genome-wide association study used by Leiboff et al.^29^, merged with additional SNPs genotyped using tGBS^30^, and those which were phased using Beagle^31^. In this paper, we extracted about 100,000 SNPs from this dataset and simulated their phenotype by combining genetic and environmental effects. In each simulation, 200 individuals were randomly selected from the 369 inbred lines to form an initial population. The purpose is to test the performance of GS algorithms using different initial breeding materials. The duration of the breeding process is set to be $T = 10$ generations. In each generation, updated genotype and phenotype data will be provided to the optimizer, which will then select 10 crosses from the current population. The simulator will simulate the creation of 20 progeny from each cross so that a constant population size of 200 is maintained throughout the breeding process. We conducted 500 independent simulations in order to account for the uncertainty in initial breeding materials and in the breeding process. For fair comparison, the same set of 500 random initial populations was used for all simulator-optimizer combinations in our experiments. We designed four simulators to compare the performances of different optimizers. Each simulator represents a possibility of nature, with S1 being the most transparent and S4 the most opaque. Simulators S1–S3 include only additive genetic effects whereas S4 also incorporates dominance and epistatic effects. In all four simulators, both genetic and environmental effects are used to determine phenotype; the environmental effects are assumed to follow a normal distribution with zero mean and a standard deviation of approximately 2% of the mean phenotype of the initial 369-line dataset.

- *Simulator S1: Transparent simulator with known allele effects* The whole genome consists of 1000 genes, all of which are assumed to have their additive effects, but no dominance or epistatic effects are assumed to exist. The optimizer is assumed to have perfect knowledge of the additive allele effects. This simulator represents a nature in which a sufficiently large number of genetic markers are used, little to no dominance effects or epistatic effects exist, and the accuracy of genomic prediction algorithm is perfect.
- *Simulator S2: Transparent simulator with unknown allele effects* The whole genome consists of 1000 genes, all of which are assumed to have their additive effects, but no dominance or epistatic effects are assumed to exist. These allele effects are unknown to the optimizer, so the genomic prediction algorithm is used to estimate them, whose accuracy depends on both the effectiveness of the algorithm and the magnitude of environmental effects. This simulator represents a nature in which a sufficiently large number of genetic markers are used, little to no dominance effects or epistatic effects exist, and the accuracy of genomic prediction algorithm is imperfect and sensitive to noisy environmental effects.
- *Simulator S3: Opaque simulator with additive effects* The whole genome consists of 100,000 genes, all of which are assumed to have their additive effects, which are unknown to the optimizer. No dominance or epistatic effects are assumed to exist. Only 1000 genetic markers are used to acquire the genotype information, and a genomic prediction algorithm is used to estimate the additive effects at these markers. This simulator represents a nature in which an insufficient number of genetic markers are used, little to no dominance effects or epistatic effects exist, and the accuracy of genomic prediction algorithm is sensitive to noisy environmental effects.
- *Simulator S4: Opaque simulator with additive and non-additive effects* The whole genome consists of 100,000 genes, all of which are assumed to have their additive effects; moreover, heterozygosity at 20 loci will trigger dominance effects, and there are 10 epistatic effects, each involving alleles at a few loci. None of these effects are unknown to the optimizer. Only 1000 genetic markers are used to acquire the genotype information, and a genomic prediction algorithm is used to estimate the additive effects at these markers. This simulator represents a nature in which an insufficient number of genetic markers are used, considerable dominance effects and epistatic effects exist, and the accuracy of genomic prediction algorithm is sensitive to non-additive genetic effects and noisy environmental effects.

**Figure 5:**
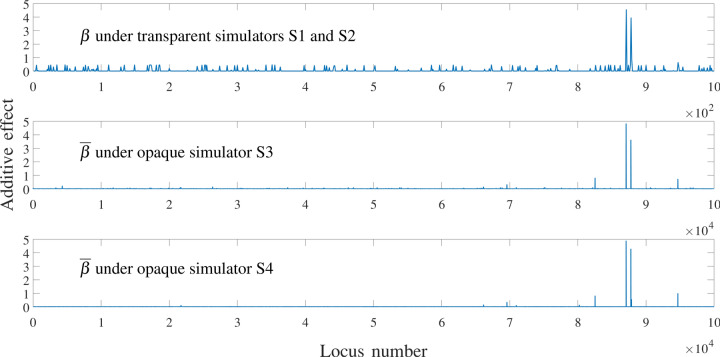
Assumed ground truth additive effects in the four simulators.

**Figure 6:**
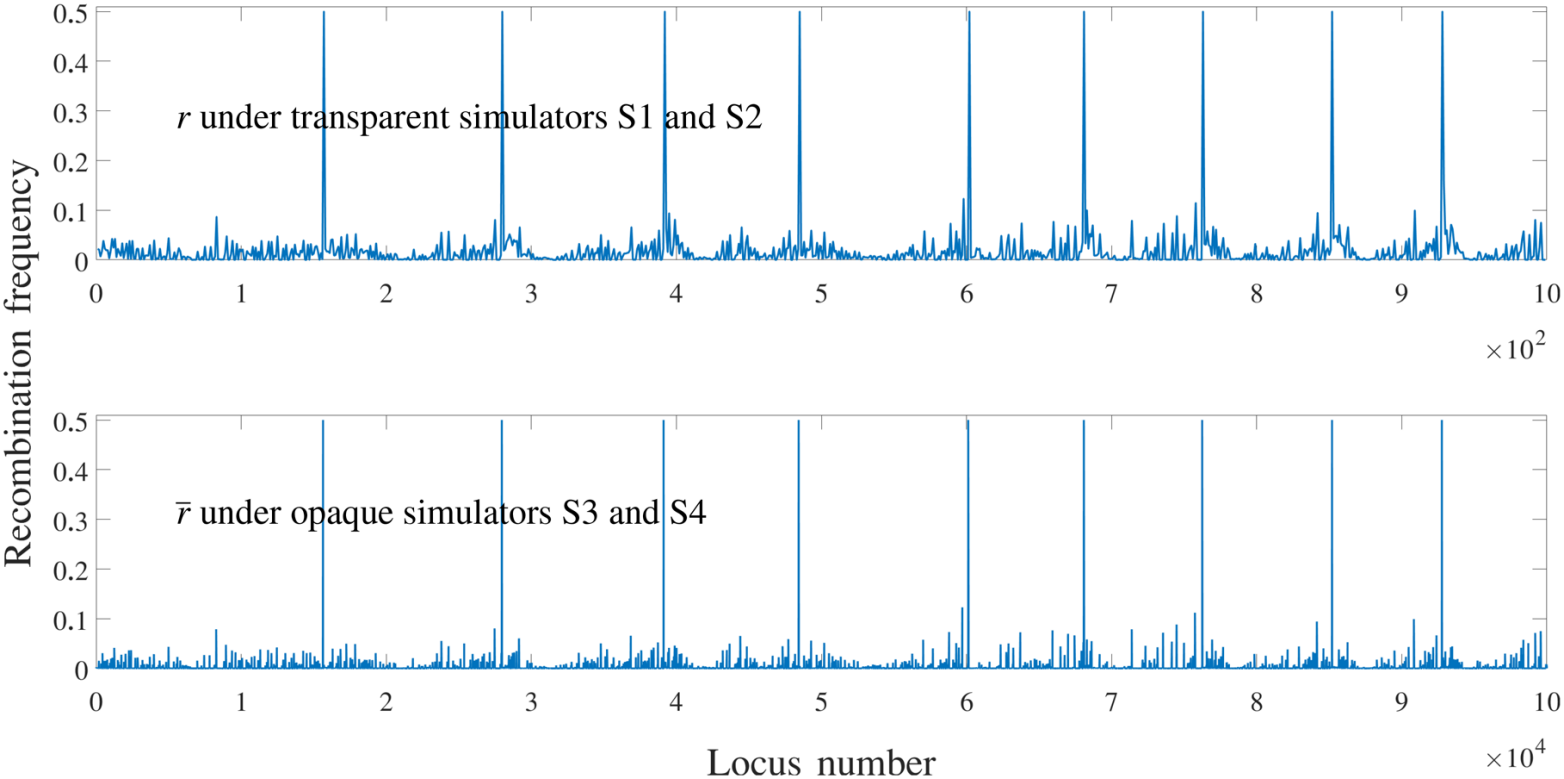
Assumed ground truth recombination frequencies in the four simulators.

The coefficients for additive ($\beta$ or $\overline{\beta }$), dominance ($\alpha$), and epistatic ($\gamma$) effects were determined to satisfy two constraints: (1) $\beta$ and $\overline{\beta }$ are non-negative vectors with $\sum _{i \in \mathcal {P}} \beta _i = \sum _{i \in \mathcal {\overline{P}}} \overline{\beta }_i = 50$ and (2) the resulting phenotype values (including additive, non-additive, and environmental effects) for the initial population of 369 lines are approximately the same under all simulators. Dominance effects $\alpha$ and epistatic effects $\gamma$ may take positive or negative values. The total additive effects in all four simulators add up to 100, which is the theoretical upper bound for simulators S1, S2, and S3; S4 may have a higher theoretical upper bound due to dominance and epistatic effects. The breakdown of phenotype of 369 lines in the initial population under four simulators are summarized in Table 1. Figure 5 shows the three $\beta$ vectors in transparent simulators S1 and S2 (top), opaque simulator S3 (middle), and opaque simulator S4 (bottom). Figure 6 shows the recombination frequency vector *r* for the transparent simulators S1 and S2 and $\overline{r}$ for the opaque simulators S3 and S4, which satisfy Eqs. (1)–(4).

**Table 1:** Breakdown of phenotype of 369 lines in the initial population under four simulators.

	S1	S2	S3	S4
Additive	$45.1 \pm 4.0$	$45.1 \pm 4.0$	$44.8 \pm 3.4$	$45.5 \pm 2.0$
Dominance	$0.0 \pm 0.0$	$0.0 \pm 0.0$	$0.0 \pm 0.0$	$0.2 \pm 2.7$
Epistatic	$0.0 \pm 0.0$	$0.0 \pm 0.0$	$0.0 \pm 0.0$	$-0.8 \pm 0.9$
Environmental	$0.0 \pm 1.0$	$0.0 \pm 1.0$	$0.0 \pm 1.0$	$0.0 \pm 1.0$
Phenotype	$45.0 \pm 4.2$	$45.0 \pm 4.1$	$44.9 \pm 3.5$	$44.9 \pm 3.4$

### Optimizer settings

#### Genomic prediction

Genomic prediction is unnecessary under simulator S1, since true allele effects are assumed to be known. Under simulators S2, S3, and S4, we use ridge regression^32,33^ to estimate the allele effect vector $\beta$ for all optimizers, so that the different outcomes can be attributed to the selection algorithms rather than the accuracy of genomic prediction. Ridge regression estimates the allele effect vector as

$$\begin{aligned} \hat{\beta }^*_k = [G^\top G + k I_p]^{-1} G^\top P, \end{aligned}$$

where $I_p$ is the $p \times p$ identity matrix and $k \in [0, 1]$ is a parameter for balancing bias and variance. It is well known that the variance of $\hat{\beta }^*_k$ is a monotonically decreasing function of *k*, which becomes zero when $k = 0$ and the model reduces to the least square estimator^34^. It has also been proven by Hoerl and Kennard^33^ that the minimum mean square error is achieved for a positive *k*, which is less than that of the least square error estimator.

In our experiments, we calculated 10 scenarios of $\hat{\beta }^*_k$ with ten different *k* values from 0 and 1. All of these $\hat{\beta }^*_k$ vectors were provided to the LATB optimizer, whereas only the one with the minimum mean square error was used in CGS and LAS optimizers.

#### PS optimizer

Selection decisions are based on the phenotypic performance. In each generation, 20 individuals with the highest phenotypes are selected and randomly mated to make 10 crosses, each producing 20 progeny.

#### CGS optimizer

Selection decisions are based on the genomic estimated breeding values (GEBVs), which are calculated using the $\beta$ vector from ridge regression as $\text {GEBV}_i = \sum _j \beta _j (G_{i,1,j} + G_{i,2,j})$. Similar to the PS optimizer, in each generation, 20 individuals with the highest GEBVs are selected and randomly mated to make 10 crosses, each producing 20 progeny.

#### LAS optimizer

The same LAS algorithm designed by Moeinizade et al.^19^ was used in the experiment. In each generation, the algorithm anticipates the performance of progeny in the final generation and then searches for the best 10 crosses to make, each producing 20 progeny.

#### LATB optimizer

The same LATB algorithm from “The LATB optimizer” was used in the experiment. In each generation, the algorithm selects the best 10 crosses, each producing 20 progeny.

### Results

**Figure 7:**
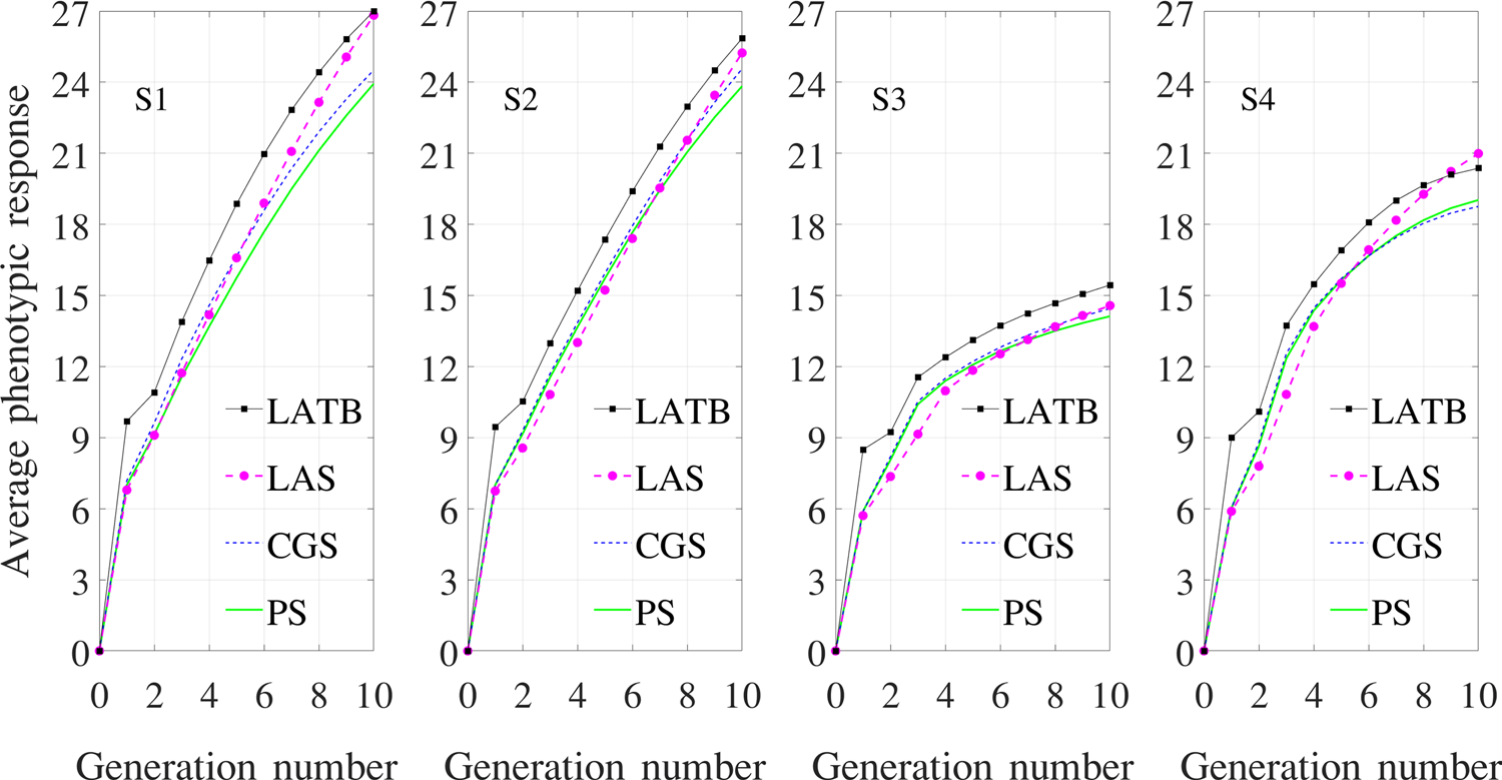
Phenotypic response over ten generations, averaged over 500 independent simulation repetitions.

Figure 7 shows the phenotypic response of the four optimizers under four simulators, averaged over 500 independent simulations. We define phenotypic response, which reflects genetic gain, for generation *t* as the difference between the average phenotype of the population in generation *t* and that for the initial generation.

**Figure 8:**
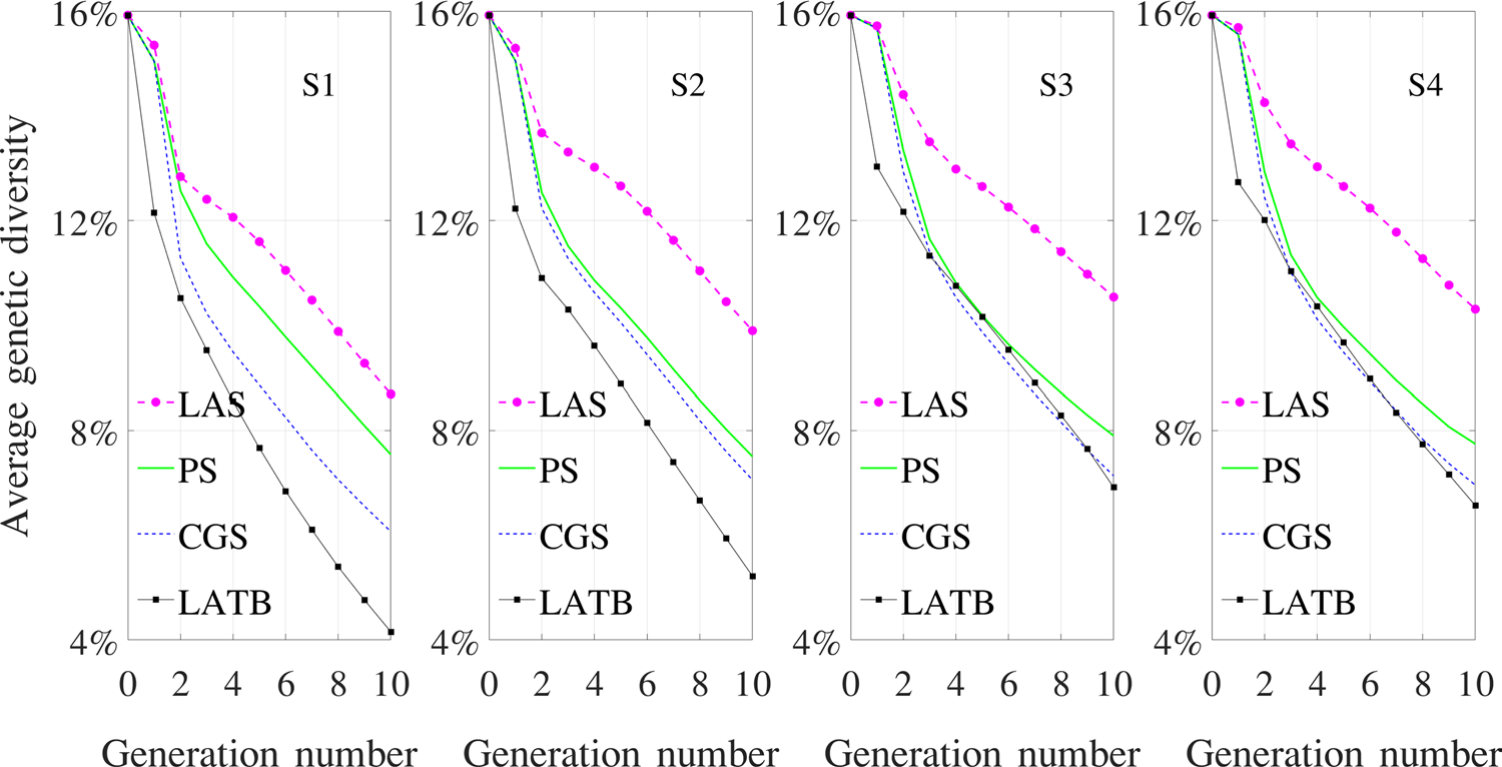
Genetic diversity over ten generations, averaged over 500 independent simulation repetitions.

Figure 8 shows the genetic diversity of the four optimizers under four simulators, averaged over 500 independent simulations. We define genetic diversity as the average frequency of rare alleles over all genetic markers. If $G \in \mathbb {B}^{n, p, 2}$ denotes the genotype of a population, then its genetic diversity is given by

$$\begin{aligned} \sum _i \dfrac{1}{n} \min \left\{ \sum _j\sum _c\dfrac{G_{i,j,c}}{2p}, 1-\sum _j\sum _c\dfrac{G_{i,j,c}}{2p}\right\} , \end{aligned}$$

where $\sum \nolimits _j\sum \nolimits _c\dfrac{G_{i,j,c}}{2p}$ is the frequency of the allele coded as “1” at locus *i*, which may or may not be rarer than the variation coded as “0”; the min operator finds the frequency of the rare allele at locus *i*; the average of such values for all genetic markers gives the genetic diversity.

## Discussions

### Performance of the four optimizers under four simulators

- PS optimizer
  - *S1 simulator* The average genetic gain makes a big leap from the initial generation of inbred lines to F1. After that, a steady increase of genetic gains is maintained throughout subsequent generations, which are all hybrids. The genetic diversity falls gradually throughout the breeding process after bigger drops in the first couple of generations.
  - *S2 simulator* The performances under S1 and S2 simulators are the same, since the knowledge of allele effects is not used by the PS optimizer.
  - *S3 simulator* The increase in average genetic gain is dramatically slower than that under the S1 and S2 simulators, which is due to the more infinitesimal assumptions of the ground truth. Recombination events at the background genes partially offset and smooth out changes at the foreground markers. The loss of genetic diversity is also slower than that under the S1 and S2 simulators, but to a much less extent compared with the genetic gain, since genetic diversity is defined for the foreground markers only.
  - *S4 simulator* The performances in both genetic gain and genetic diversity lie between S3 and S1/S2 simulators. This is intuitive because the dominance and epistatic effects would make the model less infinitesimal than S3.
- CGS optimizer
  - *S1 simulator* The average genetic gain outperforms that of the PS optimizer throughout the breeding process. This is because CGS is able to use the knowledge of the true allele effects to filter out the noisy environmental effects and select the individuals with the highest genetic values. As a result of the more accurate selection, the average genetic diversity is lost at an increasingly larger pace than the PS optimizer.
  - *S2 simulator* CGS still outdoes PS in terms of both increasing genetic gain and losing genetic diversity over time, but to a reduced extent. This is because the optimizer no longer knows the true allele effects and has to use estimated effects to select crosses, which are inevitably less effective than those under the S1 simulator.
  - *S3 simulator* Compared with PS, the average genetic gain follows almost the same trajectory before a slightly stronger finish in the tenth generation, but the average genetic diversity is lost noticeably faster. The estimated genetic effects of the partially observable genome were apparently not effective enough to filter out the random environmental effects, yet the side effect of selecting parents with similarly high genetic values still manages to manifest its erosion of genetic diversity over time.
  - *S4 simulator* The assumed existence of non-additive effects makes it even harder to estimate the true genetic values of individuals. As a result, CGS leads to a slightly lower average genetic gain in the final generation than that of PS. Estimated allele effects appear to be more helpful for selecting genetically similar parents than outstanding ones, since the average genetic diversity is lost noticeably faster than that of PS.
- LAS optimizer
  - *S1 simulator* LAS achieved a significantly higher genetic gain in the final generation than CGS while maintaining a significantly higher genetic diversity than PS. These observations are consistent with results conducted by Moeinizade et al.^19^ using an S1 type of simulator. LAS was designed to maximize genetic gain at a specific deadline without performance requirements in intermediate generations; genetic diversity was maintained as a consequence of this long-term genetic gain oriented selection strategy.
  - *S2 simulator* Using imperfect estimation of allele effects, LAS barely outperforms CGS in terms of genetic gain. The reason for this disappointment is what made LAS outstanding under S1 simulator in the first place, which is its strategy to patiently accumulate favorable alleles from a diverse population of parents, some of which may be otherwise undesirable. When the allele effects turn out to be inaccurate, expected contributions of some crosses to genetic gains may fail to materialize. On the other hand, inaccurate and changing allele effect estimates lead to more diversified selections and a higher level of genetic diversity than under the S1 simulator.
  - *S3 simulator* LAS fails to outperform CGS in genetic gain, due to not only unreliable estimate of allele effects but also the effects of 99 background genes for every 1 observable genetic marker. Genetic diversity is still significantly higher than that of PS.
  - *S4 simulator* LAS shows a surprising superiority over CGS and PS, which is comparable to that under the S1 simulator in terms of genetic gain and even more so in genetic diversity. LAS benefits from the assumed existence of genetic interactions, which creates stronger signals of dominance and epistatic effects at isolated loci for the genomic prediction algorithm to pick up, enabling LAS to exhibit its strength in accumulating desirable alleles over time. In contrast, these signals may not be as helpful to CGS, because it aggregates the estimated genetic value at the individual level rather than marker level.
- LATB optimizer
  - *S1 simulator* LATB outperforms LAS in terms of average genetic gain, but it also loses genetic diversity at a higher pace than all other optimizers. This is due to LATB’s selection strategy, which reflects the breeding process more closely than LAS. We will discuss more differences between LAS and LATB in “Differences between LATB and LAS” section.
  - *S2 simulator* LATB is still accumulating genetic gain and losing genetic diversity faster than all other optimizers, but to a discounted extent due to imperfect allele effect estimates.
  - *S3 simulator* LATB widens its superiority over other optimizers percentage wise. Since LATB was designed with unreliable allele effects in mind, it makes crosses that are less sensitive to the accuracy of genomic prediction, which explains its improvement over LAS. The decline in genetic diversity over time is only slightly faster than that of CGS.
  - *S4 simulator* Similar statements can be made as under the S3 simulator. However, a noteworthy exception is that LAS outperforms LATB in the final two generations. Since LATB uses estimated allele effects more conservatively, it does not benefit as much as LAS from the amplified signals of dominance and epistatic effects.

### Differences between LATB and LAS

First, when anticipating the consequences of crosses in the target generation, LAS assumes that all progeny will be randomly crossed with each other throughout the entire breeding process, whereas LATB explicitly expects that only the top performing progeny will be crossed with each other to produce the next generation. As a result, it is much more convenient to find the optimal set of crosses out of an enormous solution space to maximize the performance of the final generation under the LAS model, whereas the LATB model can only optimize within a relatively small subset of candidate crosses to achieve a comparable computational speed.

Second, LATB explicitly considers multiple estimates of allele effects using different prediction algorithms or parameters, which makes it less sensitive than LAS to the accuracy of the genomic prediction algorithm.

Third, the computational efficiency of LAS relies heavily on the built-in assumption of purely additive allele effects. In contrast, LATB will be compatible with more sophisticated genomic prediction algorithms that estimate both additive and non-additive allele effects, such as deep learning models^35^ and the recent algorithm for detecting epistatic effects^36^.

Fourth, computational experiment results suggest that LATB is more effective in increasing genetic gain, especially in early generations, whereas LAS maintains a higher level of genetic diversity.

### Relative importance of prediction accuracy vs. selection strategy

Performances of CGS, LAS, and LATB with respect to PS are compared in Table 2 in terms of average genetic gain and genetic diversity in the final generation. These results suggest that selection strategy has a greater impact on GS than genomic prediction accuracy.

**Table 2:** Performance comparison against PS in the final generation.

	Phenotypic response				Genetic diversity			
	S1	S2	S3	S4	S1	S2	S3	S4
PS	0%	0%	0%	0%	0%	0%	0%	0%
CGS	2%	3%	2%	− 1%	− 19%	− 6%	− 9%	− 10%
LAS	12%	6%	3%	10%	15%	31%	33%	33%
LATB	13%	9%	9%	7%	− 45%	− 31%	− 12%	− 15%

In terms of genetic gain, LAS has a much more impressive performance under S1 and S4 than under S2 and S3 simulators. In contrast, LATB is more robust: it outperforms PS by at least 7% even with imperfect genomic prediction and under the most opaque simulator. In terms of genetic diversity, LAS is the absolute winner whereas LATB makes the most compromise for genetic gain.

## Conclusions

We have presented the look ahead trace back algorithm as a new selection strategy for GS and compared its performance with other state-of-the-art approaches under multiple transparent and opaque simulators. This study made three major contributions.

First, we designed multiple versions of opaque simulators for GS, which represent different possibilities of nature. In contrast, the simulators used in most previous studies are transparent, assuming full knowledge of genotype information and purely additive allele effects. The opaque simulators were designed to capture more realistic and complex properties of nature by including partially observable genotype and non-additive genetic effects, which might not be favored by more transparent simulators. Moreover, the introduction of opacity to the simulators may motivate the invention of more robust prediction or selection algorithms.

Second, we presented the LATB algorithm as a new optimizer for making GS decisions. This algorithm attempts to improve upon the LAS algorithm by anticipating the elimination of non-elite lines in each generations and by considering imperfect prediction of allele effects. Based on the results, although LAS preserves the genetic diversity to a higher degree than LATB, LATB appears to deliver higher genetic gain within the number of cycles simulated. Furthermore, since LATB limits the crossing pool to the top-performing individuals, it shows higher initial genetic gain and facilitates the breeding process for plant breeders.

Third, we revealed the performances of four optimizers under four different simulators in comprehensive computational experiments. GS methodology performance comparisons typically rely on the use of simulation and historic datasets, however, insight that may be drawn from such comparisons is generally limited by prediction accuracy assumptions or over-estimation of prediction accuracy from widely used cross-validation methods. In this study, we demonstrated how GS methods behave differently under transparent and opaque simulators. The results highlighted the importance of designing not only efficient optimizers for GS but also realistic simulators for training and evaluating the optimizers.

Our study is not without its limitations. For example, the design of the opaque simulator may not include all complex properties of nature. The environmental effects were simply assumed to follow a normal distribution and no genotype by environment interactions were explicitly incorporated. Moreover, we found it hard to determine which of the four simulators is the closest to the nature that we live in. Ultimate validation of a simulator’s fidelity or an optimizer’s performance requires actual experiments in nature, yet our computational results shed light on the robustness and vulnerability of different optimizers under different scenarios.

Future studies should design more realistic simulators and use them to design and test more selection algorithms. Of particular interest to us is the combination of non-additive genomic prediction algorithms (such as machine learning based approaches) and the LATB algorithm. While additive effects are expected to account for the majority of the genetic variance in most traits, the optimizer’s predictive performance may be further improved by considering non-additive effects^37^.
